# Miscellaneous Intelligence

**Published:** 1801-03

**Authors:** 


### MISCELLANEOUS INTELLIGENCE.

The Royal Society of Sciences, at Gottingen, has received a prefent from Dr. Beer, oculift at Vienna, of three very mafterly drawn anatomical pathological figures of a remarkable degeneration of both kidneys, which was found in the hofpital of Vienna, in the body of a young man, who had for a long time fuffered a diabetes. The kidneys were extended by hydatids contained in their interior fubftance to fuch a monftrous fize, that each was one foot long, and feven inches broad, and the ureters were of the fize of a thumb. The chief drawing of the fore part is moft admirably coloured, and the back part excellently drawn in black chalk. The preparation itfelf is preferved in the Pathological Mufeum of the Hofpital, and Profeffor Frank intends to give a full account of this remarkable cafe.

According to the experience of Dr. Durr, the folution of muriated barytes, or terra ponderofa falita, has in fome cafes a remarkable effect upon the pulfe, and the circulation of the blood. In a fcrophulous woman of nineteen years, forty drops of a folution of muriated barytes, prepared after Hufeland's prefcription, produced a confiderable heat over the whole body, which ceafed after being leffened by degrees.—In a dofe of twenty-five or thirty drops, it caufed in a young married man nocturnal pollutions, whenever he had taken it during day-time. It is probable that this effect is owing to the diuretic powers of this remedy. (Hufeland's Practical Journal, Vol. 9, No. 3.)

The fame gentleman obferved in two cafes, that after the ceffation of the fluxus menfium, which had appeared rather diforderly and in long fpaces of time, a very ftrong fudor pedum enfued, combined with a moft difagreeable fmell which went away by degrees, as foon as the fluxus menfium was re-eftablifhed; but inftead of it an acrimony in the ftomach was brought on, which could by no remedies medies be fubdued. Dr. D. is in general of opinion, that in the inveftigation of chronical difeafes of the head and ftomach, that circumftance, *viz.* fudor pedum, is not fufficiently regarded. He relates a cafe, where, after a cuftomary fudor pedum had been repelled by bathing the warm perfpiring feet in cold water, the four back teeth of the right fide of the maxilla inferior fell out without any other perceptible caufe, and without much pain, and an efflux of a puriform matter was occafioned, which lafted a long feries of years, till the death of the patient. (*Ibid.*)

---

The New Inoculation having been ordered throughout his Majefty's Navy, by the authority of the Right Honourable the Lords Commiffioners of Admiralty, the medical officers, with becoming public fpirit, to commemorate this event, have voted a GOLD MEDAL, with appropriate devices, to be prefented in their name to Dr. JENNER; as a token of the value with which they regard his profeffional labours, and the good which mankind is likely to derive from the Vaccine practice.

*Certus enim promisit Apollo.* A Medal of exquifite workmanfhip is now executing by an eminent artift; the particulars of which, with a lift of the naval medical gentlemen, we hope to be able to prefent to our readers in fome future Number of our Journal.

---

Dr. NISBET has in the prefs a fyftematic work on DIET. It will include the application of all the modern difcoveries in chemiftry and medicine to this important fubject, and will be written in a familiar ftyle, adapted as well to the ufe of families and unprofeffional readers, as to gentlemen of the medical profeffion. Such a book has long been a defideratum, as the few books exifting which notice articles of diet, are either out of date or mixed with much extraneous and ufelefs matter.

---

Dr. BATTY will begin his Courfe of Lectures on the Theory and Practice of Midwifery, and on the Difeafes of Women and Children, on Monday, March 23, at half paft ten o'clock in the morning, at his houfe, No. 6, Great Marlborough Street.

---

Mr. MACARTNEY began a Courfe of Lectures upon Comparative Anatomy, at St. Bartholomew's Hofpital, on Thurfday the 19th of February, which will be concluded early in May.

---

Mr. FOX commenced his Lectures on the Structure and Difeafes of the Teeth, on Friday, February 7, at half paft fix o'clock. The Lectures are delivered near St. Thomas's and Guy's Hofpitals. Particulars may be known of Mr. Fox, No. 54, Lombard Street.

